# Supplementary material for: Low-voltage 2D materials-based printed field-effect transistors for integrated digital and analog electronics on paper
Source: Nat Commun. 2020 Jul 16;11:3566. doi: 10.1038/s41467-020-17297-z (PMC7367304; doi:10.1038/s41467-020-17297-z)
Supplement: Supplementary file 1 — Supplementary Information [file 41467_2020_17297_MOESM1_ESM.pdf]

Supplementary information

Low-voltage 2D materials-based printed field-effect transistors for integrated digital and analog electronics on paper

Conti *et al.*

Supplementary Note 1. CVD grown MoS_2 on SiO_2/Si substrate

Supplementary Note 1.1 Growth and transfer. As an alternative to sapphire, we have considered the CVD growth of MoS_2 on top of SiO_2 substrates. In particular, the MoS_2 flakes were obtained using the liquid precursor CVD (LPCVD) growth method [1]. First, two aqueous solutions (solution A and solution B) containing ammonium heptamolybdate (AHM, SigmaAldrich, 431346), as Mo precursor, and NaOH, as growth promoter, were prepared by dissolving 0.4 g of AHM in 30 ml deionized (DI) water and 0.1 g of NaOH in 40 ml of DI water, respectively. Then, solution A, solution B and iodixanol (Sigma-Aldrich, Opti Prep, D1556) were mixed using a ratio of 1 : 1.5 : 1 and spin coated on Si/SiO_2 substrate, which was previously cleaned in acetone, isopropanol and treated with oxygen plasma for 5 minutes. The growth was carried out in a two-zone horizontal furnace, where the spin coated substrate was heated at 700 °C in atmospheric pressure for 15 minutes under constant Ar flux, while sulphur was maintained at 200 °C. After growth, the MoS_2 flakes were transferred on paper using a semi-dry transfer approach as reported in [2]. Specifically, the sample was covered by a polymeric membrane of PMMA baked at 90 °C for 2 minutes. Then, the sample was covered using a PDMS frame and left in water at 60 °C until complete detachment of the membrane. After detachment, the free-standing membrane was transferred on the ceramic paper using a transfer set up, as previously reported [3]. Finally, the transferred sample was cleaned in acetone overnight until complete removal of PMMA.

Supplementary Note 1.2 Characterization of MoS_2 monolayers before and after transfer. Scanning Raman spectroscopy was performed with a Renishaw InVia spectrometer equipped with a confocal optical microscope and a 532 nm excitation laser. The spectral resolution of the system is 1 cm^{-1}. Raman experiments were carried out employing a 50X objective (N.A. 0.6), laser power of 5 mW and an acquisition time of 2 s. The pixel size is 1 μm x 1 μm. The Raman map of the separation of the E_{2g} and A_{1g} modes, reported in Supplementary Figure 1, indicates a highly homogenous monolayer MoS_2 crystal. It is worth noting that the black spots with a separation higher than 21 cm^{-1} can be related to bilayer terraces.

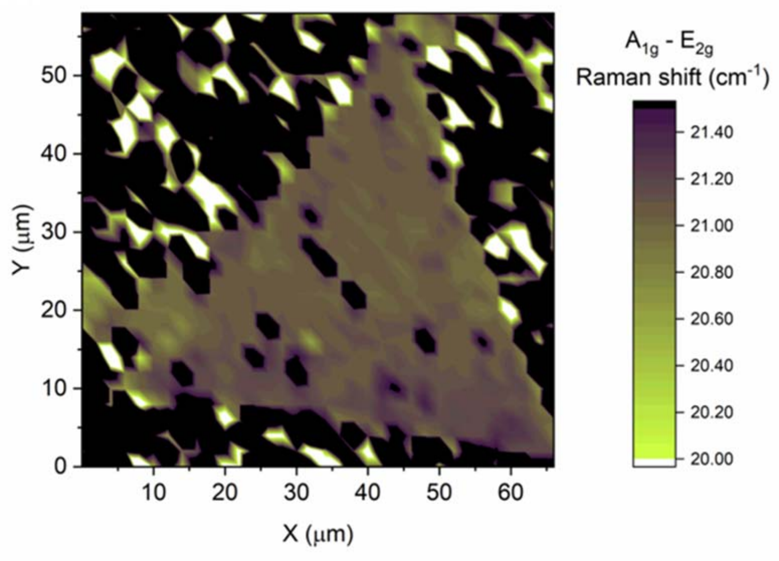

Supplementary Figure 1 | Raman map showing the separation of the E_{2g} and A_{1g} Raman modes.

Supplementary Figure 2 shows the Raman maps of A_{1g} / E_{2g} intensity ratio modes MoS_2 monolayer (ML) on the growth substrate (a) and on paper after transfer (b). The statistical analysis, reported in Supplementary Figure 2c highlights the increases of the A_{1g} / E_{2g} intensity ratio, suggesting an increase of sulphur vacancies concentration in the MoS_2 ML after the transfer process.

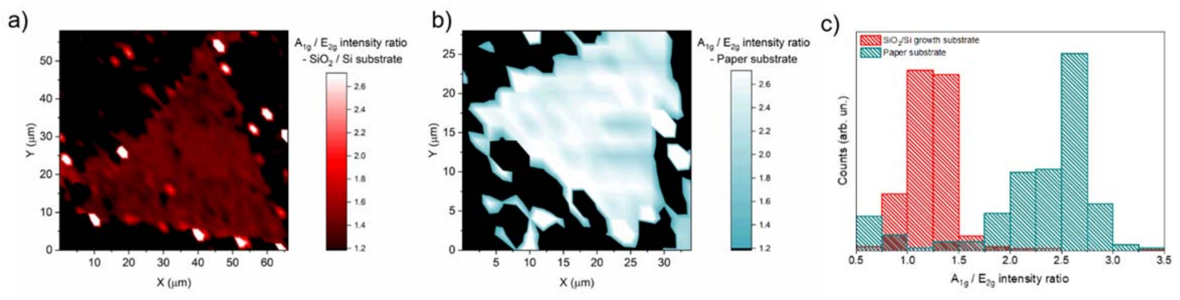

Supplementary Figure 2 | a, Raman map showing the A_{1g} /E_{2g} ratio of MoS_2 flake on the growth substrate. **b,** Raman map showing the A_{1g} /E_{2g} ratio of MoS_2 flake on paper after transfer. **c,** Statistical analysis of the A_{1g} / E_{2g} ratio before and after the transfer.

Supplementary Note 1.3 Electrical Characterization. Supplementary Figure 3a shows a single flake of MoS_2, which constitutes the device channel, in between the inkjet-printed silver source and drain contacts. The purple dotted line highlights the edges of the flake. Supplementary Figure 3b shows the complete device after printing the hBN dielectric film and the silver gate contact. The inks and the fabrication procedures for all the different components are the same as the reported in the main text (see Methods). A representative transfer characteristic curve is shown in Supplementary Figure 3c. Considering that the channel width of this transistor (W $\simeq$ 50 μm) is significantly smaller than that of the device shown in Figure 2a, b, c, d, the current densities (i.e. I_{DS}/W, where I_{DS} is the channel current) of the two devices are comparable. However, several non-idealities can be observed as, for example, a quite high I_{OFF} current. The threshold voltage is slightly negative in this case (around -1.1 V), i.e. the transistor works in depletion mode. Moreover, the mobility calculated for this transistor (0.5 $cm^2\ V^{-1}\ s^{-1}$) is smaller than the ones extracted for the other devices based on MoS_2 grown on sapphire substrate, on which the results of the main text are based on. This could be probably due to the presence of sulfur vacancies, detrimental for the transport, as highlighted through the Raman analysis.

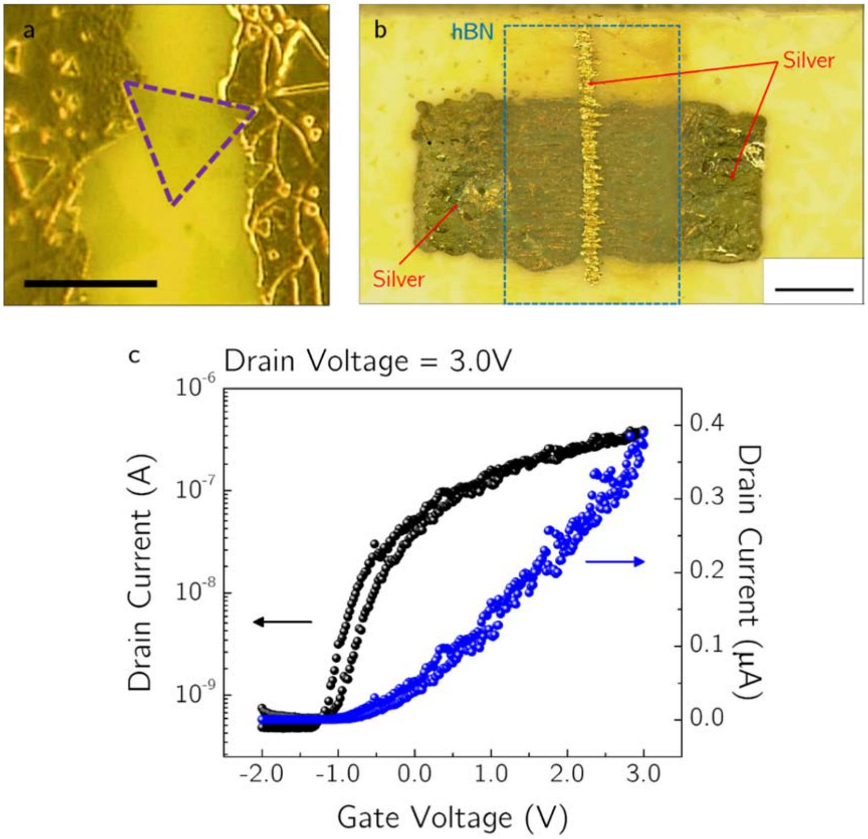

Supplementary Figure 3 | Device based on single-layer CVD grown MoS_2. a, Optical image of single flake of MoS_2 in between silver contacts. The scale bar corresponds to 50 μm. **b,** optical micrograph of the final device, after printing the hBN dielectric film and the silver gate contact. The scale bar corresponds to 250 μm. **c,** Typical transfer characteristic of a transistor based on single-layer MoS_2.

Supplementary Note 2. Atomic force microscopy of MoS_2 film on sapphire.

Supplementary Figure 4 shows an atomic force microscopy micrograph of the CVD-grown MoS_2 film, obtained from solid precursor, on sapphire before the transfer, characterized by a root mean square roughness of around 50 pm.

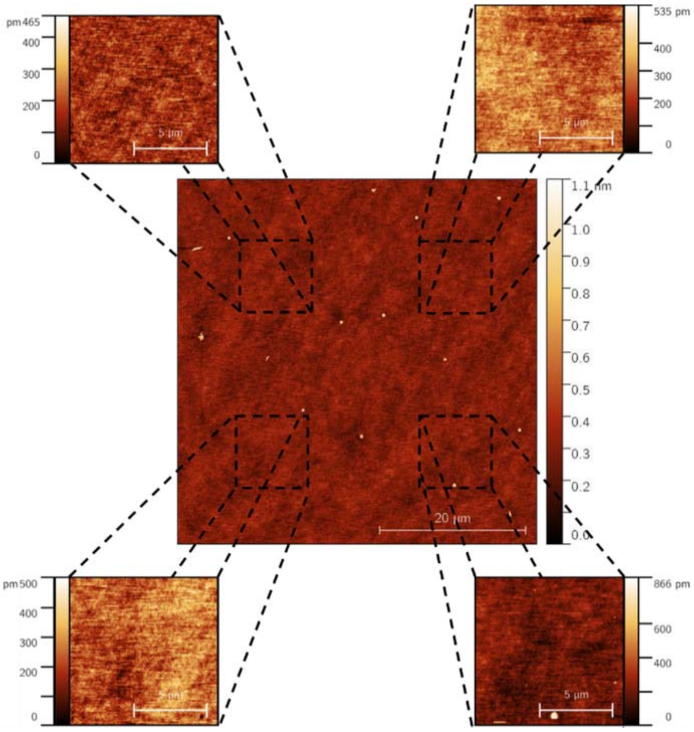

Supplementary Figure 4 | Atomic force microscopy micrograph of the CVD MoS_2. The film is characterized on sapphire before the transfer. The images on the sides are zooms of 5 µm x 5 µm of surface area.

Supplementary Note 3. Capacitance measurement.

The measurements of the insulator capacitance are carried out using parallel plate capacitor structures, as described in the main text. In order to measure the capacitance at low frequency (i.e. in quasi-static conditions), the circuit shown in Supplementary Figure 5a is used.

The capacitor under test, C, forms a capacitive voltage divider with a commercial test capacitor, C_T, with known value on the non-inverting input of an operational amplifier (Op-Amp). The Op-Amp is connected in non-inverting configuration, and has a voltage gain:

$$A_V = 1 + \frac{R_2}{R_1} \qquad \text{Supplementary Equation 1}$$

where R_1 and R_2 are two resistances, chosen to have a voltage gain of 4, in order to properly amplify the input signal. The waveform generator provides a low frequency (down to 5 mHz) sine wave V_{in}, that is applied to an input of the oscilloscope and to the capacitor under test. The voltage at the Op-Amp input, given by:

$$V^{+} = V_{in} \frac{C}{C+C_T} \qquad \text{Supplementary Equation 2}$$

is amplified to obtain the output voltage $V_{out} = A_V V^{+}$; this voltage is sent to an input of the oscilloscope. However, it is necessary to take into account that C is not an ideal capacitor; in order to have a more realistic model, the capacitor is represented with the equivalent circuit shown in Supplementary Figure 5b, according to [4] C_p represents the capacitive part of the capacitor, while the resistor R_p takes into account the dielectric leakage. Therefore, the output voltage is modified as follows:

$$V_{out} = A_V \frac{R_p C_p s+1}{R_p(C_T+C_p)s+1} V_{in} = H(s)V_{in} \qquad \text{Supplementary Information 3}$$

where s is the Laplace generalized frequency and $H(s)$ is the transfer function between V_{in} and V_{out}. Supplementary Figure 5c shows the voltages V_{in} and V_{out} measured with the oscilloscope. The phase shift φ_0 between the two sine waves, highlighted in Supplementary Figure 5d, is due to the presence of the resistance R_p.

The measurement of the output voltage amplitude and the phase shift φ_0, i.e. of the module and phase of the transfer function $H(f)$ (f is the frequency), allows to solve the system of non-linear equations:

$$\begin{cases} |H(f)| = \dfrac{\sqrt{R_p{}^2 C_p{}^2 (2\pi f)^2+1}}{\sqrt{R_p{}^2 (C_T+C_p)^2 (2\pi f)^2+1}} \\ \angle H(f) = atan\big(R_p C_p 2\pi f\big) - atan\big(R_p (C_T + C_p) 2\pi f\big) \end{cases} \qquad \text{Supplementary Equation 4}$$

and, therefore, to obtain the resistance R_p and the capacitance C_p. The insulator areal capacitance is obtained dividing the extracted capacitance value by the area of the test capacitor.

The extracted average value of 230 nF cm^{-2} explains the low threshold voltages of the devices. In quasi-static conditions, the presence of both water molecules (water is the solvent of the hBN ink employed in this work [5]) and impurities (moisture absorbance, mobile ions) in the films could influence the charging processes thus increasing its permittivity, as widely observed for both organic and hybrid materials [6,7,8,9,10].

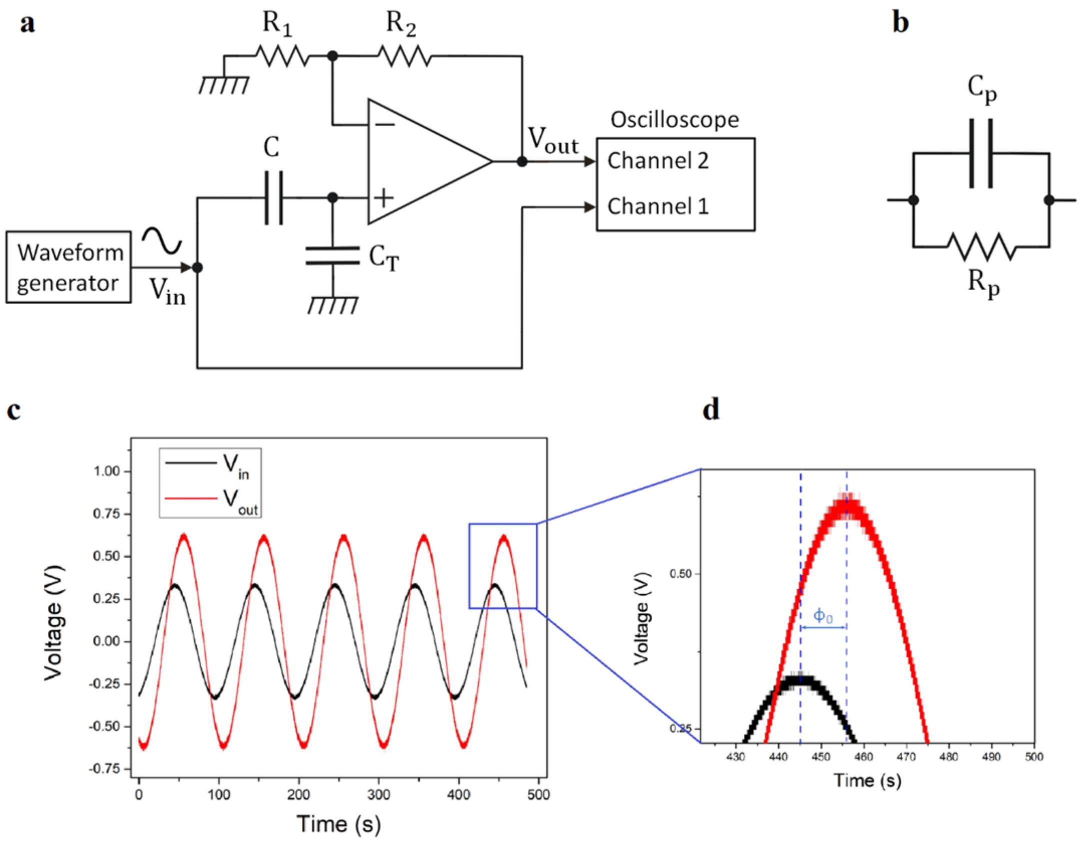

Supplementary Figure 5 | Capacitance measurement setup. a, schematic of the electronic circuit used for the measurement of the capacitance. **b,** equivalent circuit of the capacitor under measurement. **c,** input and output voltages as a function of time. **d,** magnification of the measured signals showing the phase shift between the input and output voltages.

Supplementary Note 4. Detailed electrical characterization.

The values of threshold voltage and field-effect mobility reported in the main text are evaluated using the following procedures. Supplementary Figure 6 shows the square root of the channel current as a function of the gate voltage for a representative device. The value of threshold voltage is extrapolated as the *x* axis intercept of the straight line fitting the square root of the drain current, as shown in the figure. The field-effect mobility has been extracted using the formula in saturation regime [equation (1) of the main text].

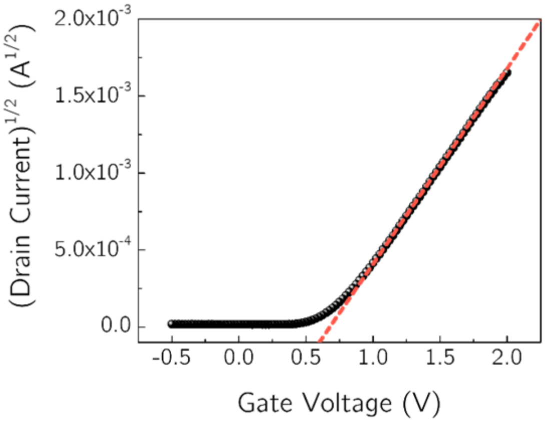

Supplementary Figure 6 | Extraction of the MoS2 FET electrical parameters. Square root of the drain current as a function of the gate voltage. Fit line in red illustrates the range used to fit the data and extract the threshold voltage and the filed-effect mobility value.

In Supplementary Figure 7 the electrical characterization of 26 MoS_2 FETs is reported. Supplementary Figure 7a and Supplementary Figure 7b show the transfer characteristic curves and the gate current vs gate voltage curves for each devices, respectively. A yield of 80% was obtained. As a criterion, we have excluded devices with I_{Leak} > 5 nA. A possible explanation for the high I_{Leak} observed in some devices, is likely related to non-uniformities in the insulating layers, which can lead to the presence of pinholes in the printed film [11].

The distribution of the threshold voltages and the field-effect mobility values in the forward sweep are reported Supplementary Figure 7c and Supplementary Figure 7d, respectively. The distribution of the threshold voltages and the field-effect mobility values in the backward sweep are reported Supplementary Figure 7e and Supplementary Figure 7f, respectively. The variability of the threshold voltages and the mobility values are, in general, a function of the dielectric, the semiconductor, and their interface [12]. The on currents vary from 1 μA to nearly 50μA. This is probably related to inhomogeneities in the CVD MoS_2 films and the presence of MoS_2 grain boundaries [13]. Charge carriers interacting with shallow traps, possibly at the MoS_2/dielectric interface can be the cause, of the anticlockwise hysteresis shown in Supplementary Figure 7a and,

consequently, of the variation in the threshold voltage (the average value $V_{THforward}$ is (0.38 ± 0.07) V, the average value $V_{THbackward}$ is (0.14 ± 0.06) V.

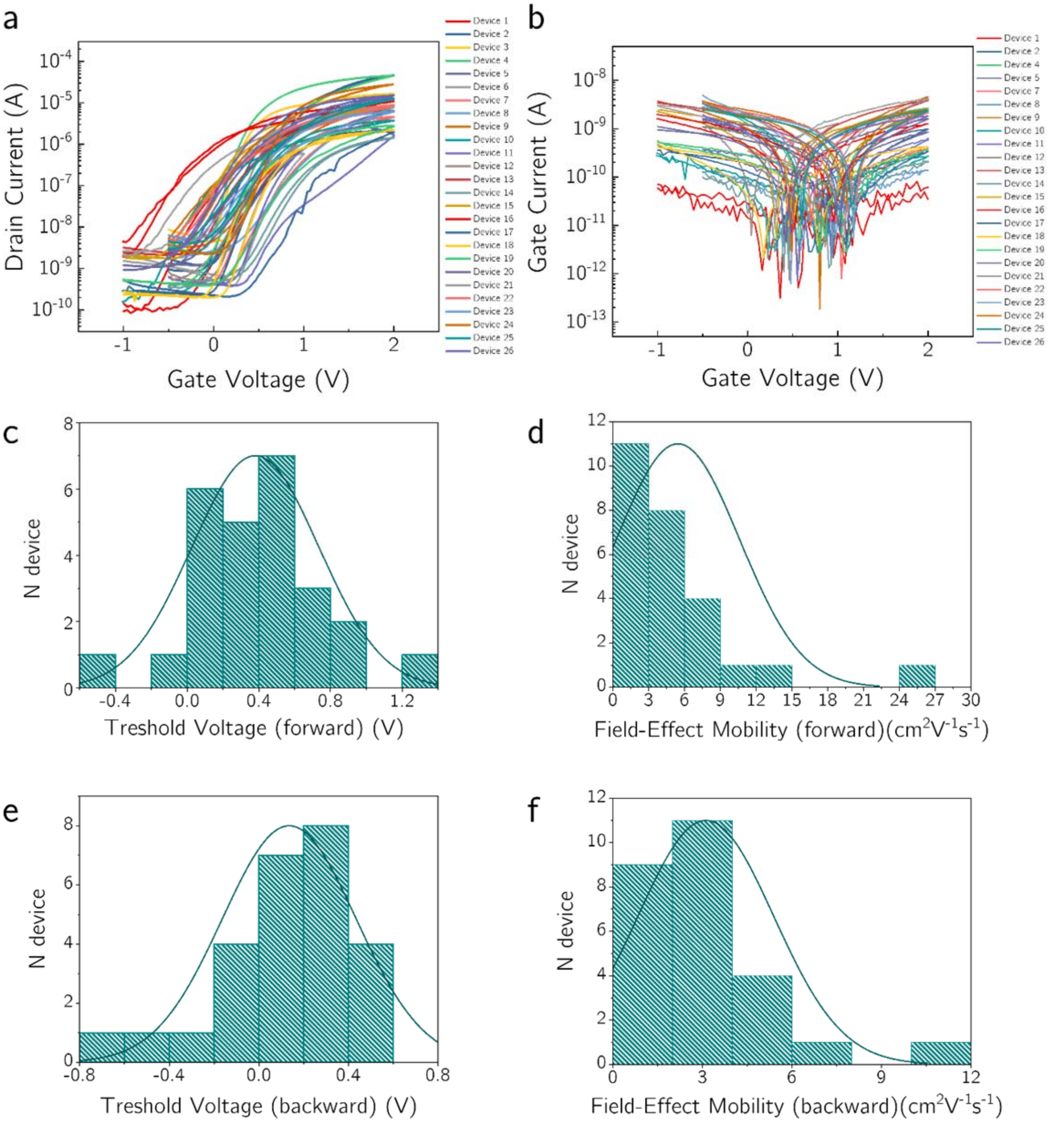

Supplementary Figure 7 | Electrical characterization of 26 MoS_2 FETs. a, Transfer characteristic curves. b, Gate current vs gate voltage curve. c, Distribution of treshold voltage values (forward). d, Distribution of field-effect mobility values (forward). e, Distribution of treshold voltage values (backward). f, Distribution of field-effect mobility values (backward).

Supplementary Note 4.1 Bending test. To test the devices under tensile strain conditions, the paper substrate is wrapped around rigid jigs of different radii (R: 32 mm, 20 mm, 12 mm, and 8 mm) and the electrical performance are characterized using the same setup (probe tips, ambient condition) reported in Methods. The tensile strain *S* to the bent channel can be calculated using the following equation [13,14]:

$$S = \frac{t_{MoS_2} + t_{total}}{2R} x\, 100 \qquad \text{Supplementary Equation 5}$$

Where t_{MoS2}, t_{total}, and R are the thickness of the MoS_2 layer, the thickness of the device, and the bending radius, respectively. The paper employed in this work is characterized by a thickness of 275 μm; thus, t_{total} can be considered equal to the substrate thickness. Tensile strains of 0.43%, 0.69%, 1.15%, and 1.72% are obtained from Supplementary Equation 5.

Supplementary Note 5. Comparison with MoS_2-based FETs on flexible substrates.

Supplementary Figure 8 shows μ_{FE} and $(I_{ON}/I_{OFF})/V_{DD}$ for our devices compared with those previously reported in the literature: the closer the points to the top-right corner, the better the performance. MoS_2-based transistors fully fabricated on flexible substrates or transferred on flexible substrates after fabrication are considered. The I_{ON}/I_{OFF} values are re-calculated considering the ITRS definition [15], and then divided by V_{DD}. Supplementary Data 2 reports the substrates, materials and deposition techniques, V_{GS} supply, μ_{FE}, and $(I_{ON}/I_{OFF})/V_{DD}$ for the manuscripts reported in Figure S8. The devices have been divided into 3 groups, according to the MoS_2 fabrication process. The key challenge for the development of high-performing flexible electronics is the use of optimal semiconductors that show good mechanical flexibility, that can be processed using low-temperature approaches, and, most importantly, exploited in large-scale integrated circuits [33]. For these reasons, devices in the grey area [33,34,35,36,37,38,39], which all present remarkable performances and are fabricated using mechanical exfoliated MoS_2, cannot be considered as a valid option. Although devices in the green area don't show competitive performances, they have been included in this graph because they are all fabricated using quite challenging deposition techniques for the semiconductor [17,40,41,42,43]. In 2017, for example, Kelly *et al.* [40], demonstrated a low-voltage FET characterized by a mobility of 0.15 $cm^2\ V^{-1}\ s^{-1}$ and I_{ON}/I_{OFF} value of around 20, using spray coating for the deposition of the active layer. All the transistors reported in the pink area are fabricated with CVD MoS_2 semiconducting layers [13,16,44,45,46,47,48,49,50,51,52,53]. As can be seen, our devices are well placed in terms of performance as compared to the others, and they are the only ones that simultaneously present inkjet-printed insulating and contacts layers, are fabricated on paper, and can be operated using low-voltages.

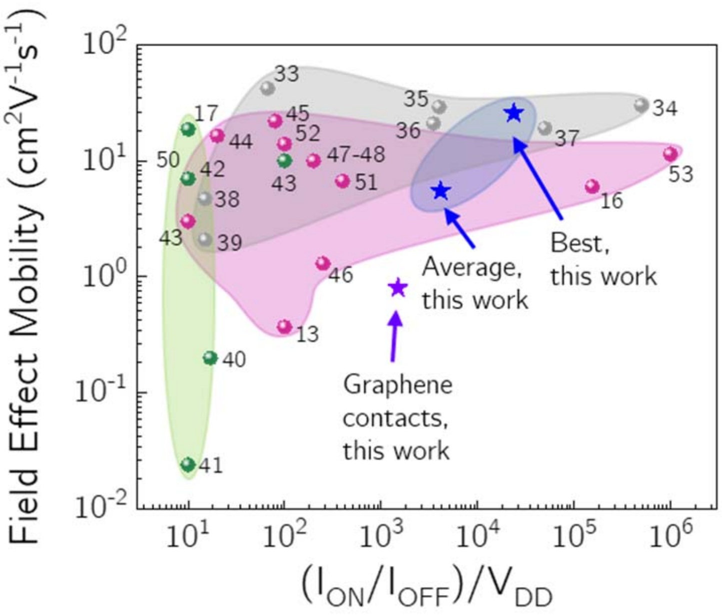

Supplementary Figure 8 | Field-effect mobility and $(I_{ON}/I_{OFF})/V_{DD}$ for MoS_2-based FETs characterized on flexible substrates previously reported in the literature. V_{DD} is the supply voltage for each device. Blue stars, this work, inkjet-printed silver contacts; purple star, this work, inkjet-printed graphene contacts; grey dots, mechanical exfoliated MoS_2 (33, 34, 35, 36, 37, 38, 39); green dots, other deposition methods (17, 40, 41, 42, 43); fuchsia dots, CVD MoS_2 (13, 16, 44, 45, 46, 47, 48, 49, 50, 51, 52, 53). A detail comparison is reported in Supplementary Data 2.

Supplementary Note 6. PEL P60 details.

PEL P60 (purchased from Printed Electronics Limited) is a cellulose-based paper (of a thickness of around 250 μm) coated with a microporous ceramic slurry (of a thickness of around 25 μm). Supplementary Figure 9 shows the profile of the paper surface.

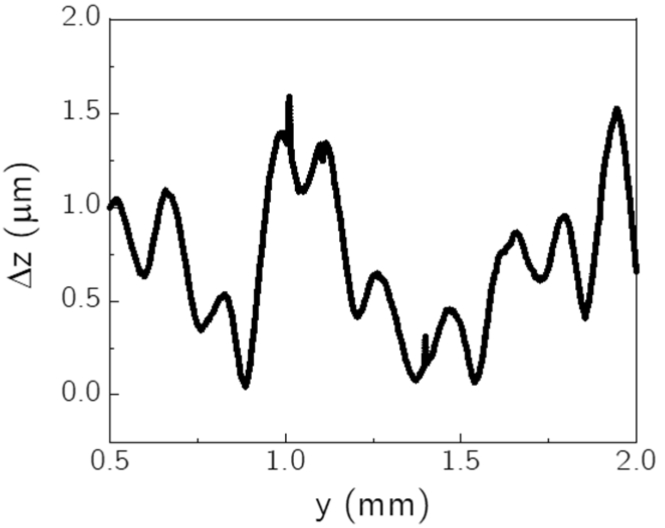

Supplementary Figure 9 | Variation of the surface of the paper substrate employed in this work.

Supplementary Note 7. FET geometrical dimensions.

Supplementary Table 1 | Widths and lengths of 26 MoS_2 FETs.

Device number	Width (µm)	Lenght (µm)
Device 1	505	55
Device 2	370	40
Device 3	460	50
Device 4	470	50
Device 5	500	55
Device 6	500	55
Device 7	500	50
Device 8	510	55
Device 9	250	30
Device 10	250	30
Device 11	500	50
Device 12	500	55
Device 13	505	50
Device 14	500	55
Device 15	500	55
Device 16	500	50
Device 17	510	55
Device 18	500	55
Device 19	500	60
Device 20	510	50
Device 21	505	50
Device 22	490	50
Device 23	500	50
Device 24	515	40
Device 25	500	50
Device 26	505	50

Supplementary References

1. Kim, H. *et al.* Role of alkali metal promoter in enhancing lateral growth of monolayer transition metal dichalcogenides. *Nanotechnology* **28**, 36LT01 (2017).

2. Miseikis, V. *et al.* Deterministic patterned growth of high-mobility large-crystal graphene: A path towards wafer scale integration. *2D Mater.* **4**, (2017).

3. Miseikis, V. *et al.* Rapid CVD growth of millimetre-sized single crystal graphene using a cold-wall reactor. *2D Mater.* **2**, (2015).

4. Worsley, R. *et al.* All-2D Material Inkjet-Printed Capacitors: Toward Fully Printed Integrated Circuits. *ACS Nano* **13**, 54–60 (2019).

5. McManus, D. *et al.* Water-based and biocompatible 2D crystal inks for all-inkjet-printed heterostructures. *Nat. Nanotechnol.* **12**, 343–350 (2017).

6. Bäcklund, T. G., Österbacka, R., Stubb, H., Bobacka, J. & Ivaska, A. Operating principle of polymer insulator organic thin-film transistors exposed to moisture. *J. Appl. Phys.* **98**, 074504 (2005).

7. Kim, S. H. *et al.* Effect of the hydrophobicity and thickness of polymer gate dielectrics on the hysteresis behavior of pentacene-based field-effect transistors. *J. Appl. Phys.* **105**, 104509 (2009).

8. Kong, D. *et al.* Capacitance Characterization of Elastomeric Dielectrics for Applications in Intrinsically Stretchable Thin Film Transistors. *Adv. Funct. Mater.* **26**, 4680–4686 (2016).

9. Zhang, C. X. *et al.* Total ionizing dose effects on hBN encapsulated graphene devices. *IEEE Trans. Nucl. Sci.* **61**, 2868–2873 (2014).

10. Tsekmes, I. A., Kochetov, R., Morshuis, P. H. F. & Smit, J. J. Evaluating the effect of particle distribution and dispersion on the dielectric response of boron nitride - epoxy nanocomposites. in *2014 IEEE Electrical Insulation Conference (EIC)* 329–332 (IEEE, 2014).

11. Kelly, A. G., Finn, D., Harvey, A., Hallam, T. & Coleman, J. N. All-printed capacitors from graphene-BN-graphene nanosheet heterostructures. *Appl. Phys. Lett.* **109**, 023107 (2016).

12. Feng, W., Zheng, W., Cao, W. & Hu, P. Back Gated Multilayer InSe Transistors with Enhanced Carrier Mobilities via the Suppression of Carrier Scattering from a Dielectric Interface. *Adv. Mater.* **26**, 6587–6593 (2014).

13. Kim, T.-Y. *et al.* Transparent Large-Area MoS_2 Phototransistors with Inkjet-Printed Components on Flexible Platforms. *ACS Nano* **11**, 10273–10280 (2017).

14. Casiraghi, C. *et al.* Inkjet printed 2D-crystal based strain gauges on paper. *Carbon N. Y.* **129**, 462–467 (2018).

15. International Technology Roadmap for Semiconductors - ITRS. Available at: http://www.itrs2.net/.

16. Park, S. & Akinwande, D. First demonstration of high performance 2D monolayer transistors on paper substrates in *2017 IEEE International Electron Devices Meeting (IEDM)* 5.2.1-5.2.4 (IEEE, 2017).

17. Sahatiya, P. & Badhulika, S. Wireless, Smart, Human Motion Monitoring Using Solution Processed Fabrication of Graphene-MoS_2 Transistors on Paper. *Adv. Electron. Mater.* **4**,

1700388 (2018).

18. Veeralingam, S. & Badhulika, S. 2D - $SnSe_2$ nanoflakes on paper with 1D - NiO gate insulator based MISFET as multifunctional NIR photo switch and flexible temperature sensor. *Mater. Sci. Semicond. Process.* **105**, 104738 (2020).

19. Huang, J. *et al.* Highly Transparent and Flexible Nanopaper Transistors. *ACS Nano* **7**, 2106–2113 (2013).

20. Minari, T. *et al.* Room-temperature printing of organic thin-film transistors with π-junction gold nanoparticles. *Adv. Funct. Mater.* **24**, 4886–4892 (2014).

21. Fujisaki, Y. *et al.* Transparent Nanopaper-Based Flexible Organic Thin-Film Transistor Array. *Adv. Funct. Mater.* **24**, 1657–1663 (2014).

22. Hyun, W. J. *et al.* All-Printed, Foldable Organic Thin-Film Transistors on Glassine Paper. *Adv. Mater.* **27**, 7058–7064 (2015).

23. Dai, S. *et al.* Intrinsically ionic conductive cellulose nanopapers applied as all solid dielectrics for low voltage organic transistors. *Nat. Commun.* **9**, 2737 (2018).

24. Kraft, U. *et al.* Low-Voltage, High-Frequency Organic Transistors and Unipolar and Complementary Ring Oscillators on Paper. *Adv. Electron. Mater.* **5**, 1800453 (2019).

25. Casula, G. *et al.* Printed, Low-Voltage, All-Organic Transistors and Complementary Circuits on Paper Substrate. *Adv. Electron. Mater.* **1901027**, 1–9 (2020).

26. Martins, R. F. P. *et al.* Recyclable, Flexible, Low-Power Oxide Electronics. *Adv. Funct. Mater.* **23**, 2153–2161 (2013).

27. Thiemann, S. *et al.* Cellulose-based ionogels for paper electronics. *Adv. Funct. Mater.* **24**, 625–634 (2014).

28. Kim, S. J. *et al.* Nonvolatile memory thin-film transistors using biodegradable chicken albumen gate insulator and oxide semiconductor channel on eco-friendly paper substrate. *ACS Appl. Mater. Interfaces* **7**, 4869–4874 (2015).

29. Gaspar, D. *et al.* Planar Dual□Gate Paper/Oxide Field Effect Transistors as Universal Logic Gates. *Adv. Electron. Mater.* **4**, 1800423 (2018).

30. Wang, X., Gao, Y., Liu, Z., Luo, J. & Wan, Q. Flexible Low-Voltage IGZO Thin-Film Transistors With Polymer Electret Gate Dielectrics on Paper Substrates. *IEEE Electron Device Lett.* **40**, 224–227 (2019).

31. Liu, N., Yun, K. N., Yu, H.-Y., Shim, J. H. & Lee, C. J. High-performance carbon nanotube thin-film transistors on flexible paper substrates. *Appl. Phys. Lett.* **106**, 103106 (2015).

32. Yoon, J. *et al.* Flammable carbon nanotube transistors on a nitrocellulose paper substrate for transient electronics. *Nano Res.* **10**, 87–96 (2017).

33. Chung, J. W. *et al.* Flexible nano-hybrid inverter based on inkjet-printed organic and 2D multilayer MoS_2 thin film transistor. *Org. Electron.* **15**, 3038–3042 (2014).

34. Chang, H. Y. *et al.* High-performance, highly bendable MoS_2 transistors with high-K dielectrics for flexible low-power systems. *ACS Nano* **7**, 5446–5452 (2013).

35. Lee, G. H. *et al.* Flexible and transparent MoS_2 field-effect transistors on hexagonal boron nitride-graphene heterostructures. *ACS Nano* **7**, 7931–7936 (2013).

36. Lee, I. *et al.* Ultrahigh Gauge Factor in Graphene/MoS_2 Heterojunction Field Effect Transistor with Variable Schottky Barrier. *ACS Nano* **13**, 8392–8400 (2019).

37. Salvatore, G. A. *et al.* Fabrication and transfer of flexible few-layers MoS_2 thin film transistors to any arbitrary substrate. *ACS Nano* **7**, 8809–8815 (2013).

38. Yoon, J. *et al.* Highly Flexible and Transparent Multilayer MoS_2 Transistors with Graphene Electrodes. *Small* **9**, 3295–3300 (2013).

39. Lee, H. *et al.* Transfer of transition-metal dichalcogenide circuits onto arbitrary substrates for flexible device applications. *Nanoscale* **11**, 22118–22124 (2019).

40. Kelly, A. G. *et al.* All-printed thin-film transistors from networks of liquid-exfoliated nanosheets. *Science.* **356**, 69–73 (2017).

41. Sirota, B., Glavin, N. & Voevodin, A. A. Room temperature magnetron sputtering and laser annealing of ultrathin MoS_2 for flexible transistors. *Vacuum* **160**, 133–138 (2019).

42. Tsai, W.-C. & Lin, I.-C. Development of a piezoelectric immunosensor for the detection of alpha-fetoprotein. *Sensors Actuators B Chem.* **106**, 455–460 (2005).

43. Lin, Z. *et al.* Solution-processable 2D semiconductors for high-performance large-area electronics. *Nature* **562**, 254–258 (2018).

44. Amani, M., Burke, R. A., Proie, R. M. & Dubey, M. Flexible integrated circuits and multifunctional electronics based on single atomic layers of MoS_2 and graphene. *Nanotechnology* **26**, (2015).

45. Chang, H. Y. *et al.* Large-Area Monolayer MoS_2 for Flexible Low-Power RF Nanoelectronics in the GHz Regime. *Adv. Mater.* **28**, 1818–1823 (2016).

46. Das, T. *et al.* Highly Flexible Hybrid CMOS Inverter Based on Si Nanomembrane and Molybdenum Disulfide. *Small* **12**, 5720–5727 (2016).

47. Jang, J. *et al.* Mechanoluminescent, Air-Dielectric MoS 2 Transistors as Active-Matrix Pressure Sensors for Wide Detection Ranges from Footsteps to Cellular Motions. *Nano Lett.* **20**, 66–74 (2020).

48. Petrone, N., Cui, X., Hone, J., Chari, T. & Shepard, K. Flexible 2D FETs using hBN dielectrics. *Tech. Dig. - Int. Electron Devices Meet. IEDM*, 19.8.1-19.8.4 (2015).

49. Pu, J. *et al.* Highly flexible MoS_2 thin-film transistors with ion gel dielectrics. *Nano Lett.* **12**, 4013–4017 (2012).

50. Qu, Z., Tang, H., Ye, H., Fan, X. & Zhang, G. Electrical and optical characterization of MoS_2 thin film transistors and the effect of strain on their performances. in *2019 20th International Conference on Thermal, Mechanical and Multi-Physics Simulation and Experiments in Microelectronics and Microsystems (EuroSimE)* 1–5 (IEEE, 2019).

51. Shinde, S. M. *et al.* Surface-Functionalization-Mediated Direct Transfer of Molybdenum Disulfide for Large-Area Flexible Devices. *Adv. Funct. Mater.* **28**, 1–11 (2018).

52. Zhao, J. *et al.* Integrated Flexible and High-Quality Thin Film Transistors Based on Monolayer MoS_2 . *Adv. Electron. Mater.* **2**, 1500379 (2016).

53. Zhu, Y. *et al.* Monolayer Molybdenum Disulfide Transistors with Single-Atom-Thick Gates. *Nano Lett.* **18**, 3807–3813 (2018).
