# Supplementary material for: Low-voltage 2D materials-based printed field-effect transistors for integrated digital and analog electronics on paper
Source: Nat Commun. 2020 Jul 16;11:3566. doi: 10.1038/s41467-020-17297-z (PMC7367304; doi:10.1038/s41467-020-17297-z)
Supplement: Supplementary file 2 — Descriptions of Additional Supplementary Files [file 41467_2020_17297_MOESM2_ESM.pdf]

Descriptions of Additional Supplementary Files

Supplementary Data 1

Description: Comparison with FETs on paper substrates. Literature table showing the reference number, materials and deposition techniques, gate-to-source voltage (VGS) supply, field-effect mobility (µFE), and (ION/IOFF)VDD for the manuscripts reported in Figure 2e of the main text. ION/IOFF values have been recalculated according to the ITRS definition 15. VDD is the supply voltage. The reference numbers are the ones from the main text. The range of the applied gate voltage is classified as high voltage (HV, above 10 V) or low voltage (LV, below 10 V). G, gate electrode; S/D, source/drain electrode; where not specified the same material is used for all the electrodes.

Supplementary Data 2

Description: Comparison with MoS2-based FETs on flexible substrates. Literature table for Figure S8. Literature table showing reference numbers, substrates, materials and deposition techniques, VGS supply, µFE, and (ION/IOFF)/VDD for the manuscripts reported in Figure S8. ION/IOFF values have been recalculated according to the ITRS definition S15. The reference numbers are referred to the numeration reported in the supplementary information. The range of the applied gate voltage is classified as high voltage (HV, above 10 V) or low voltage (LV, below 10 V). G, gate electrode; S/D, source/drain electrodes; where not specified the same material is used for all electrodes. Polymide (PI); polyethylene terephthalate (PET); polyethylene−naphthalate (PEN); polydimethylsiloxane (PDMS); 1-Ethyl-3-methylimidazolium bis(trifluoromethylsulfonyl)imide (EMIm TFSI); poly(acrylic acid (PAA); liquid phase epitaxy (LPE); plasma enhanced CVD (PECVD); low pressure CVD (LPCVD).
